# Characteristics of Prior Authorization Policies for New Drugs in Medicare Part D

**DOI:** 10.1001/jamahealthforum.2022.5610

**Published:** 2023-02-24

**Authors:** Huseyin Naci, Robin Forrest, Mike Zhai, Amanda R. Stofesky, Aaron S. Kesselheim

**Affiliations:** 1 Department of Health Policy, London School of Economics and Political Science, London, United Kingdom; 2 Program On Regulation, Therapeutics, And Law (PORTAL), Division of Pharmacoepidemiology and Pharmacoeconomics, Department of Medicine, Brigham and Women’s Hospital, Harvard Medical School, Boston, Massachusetts; 3 Dartmouth Institute for Health Policy and Clinical Practice, Dartmouth College, Lebanon, New Hampshire

## Abstract

This cross-sectional study examines the characteristics of prior authorization policies for new drugs in Medicare Part D to understand whether they are consistent with US Food and Drug Administration indications.

## Introduction

Health insurance providers in the US can employ prior authorization (PA), for which approval from the insurer is needed before a prescription can be dispensed. Prior authorization is most prevalent among expensive or complex therapies to ensure appropriate use. Physicians report that PA can be administratively burdensome.^1,2^

In Medicare Part D, drug benefits are administered by a multitude of private plans, each with its own formulary and benefit design. An earlier study found that most new drugs covered in Part D are subject to PA requirements, primarily due to high launch prices.^3^ However, characteristics of such requirements have not been examined. We sought to describe the characteristics of PA policies for new drugs in Part D to understand whether they are consistent with US Food and Drug Administration (FDA) indications.

## Methods

We used Drugs@FDA to identify all new drugs approved from 2013 to 2017. We determined whether the drugs were included in plan formularies administered by 8 insurers that covered almost 90% of Medicare beneficiaries enrolled in Part D.^4^ Because PA policies are consistent across plans offered in different states, we identified drug plans offered by each insurer in 2020 in Cambridge, Massachusetts (zip code 02139). We separately searched for Kaiser Permanente’s plan in Los Angeles, California (zip code 90029).

For each insurer, we reviewed the summary of benefits files and PA policies. For PA drugs, we extracted information on 6 descriptive categories (exclusion criteria; required medical information; age restrictions; prescriber restrictions; coverage duration limitations; and other) that were required by the Centers for Medicare & Medicaid Services (CMS) to be reported in PA documentation (see the eAppendix in Supplement 1 for data extraction process).

Two investigators (H.N. and R.F.) compared the PA information to the drug’s FDA-approved indication, noting if plans mirrored the approved labeling or were more restrictive. Any restrictions were confirmed by another investigator (M.Z.). We counted the number of drugs for which coverage was conditional on PA in at least 1 plan and the number of drugs covered by at least 1 plan with PA more restrictive than the approved labeling.

The study was deemed exempt from institutional review board approval according to 45 CFR §46.102 because it used public, nonidentifiable data and did not constitute human participants research.

## Results

Among 109 drugs eligible for Part D coverage, most (104 [95%]) were on the formulary of at least 1 of the largest insurers in 2020. Two-thirds of drugs on at least 1 Part D formulary (72 [66%]) were covered with PA. Across the 8 insurers covering 638 total PA policies, the most common category of PA was submitting medical information (254 [39%]). There was substantial variation in the frequency and type of PAs implemented by different plans (Figure). The total number of requirements ranged from 8 (Kaiser Permanente) to 187 (Humana).

**Figure.**
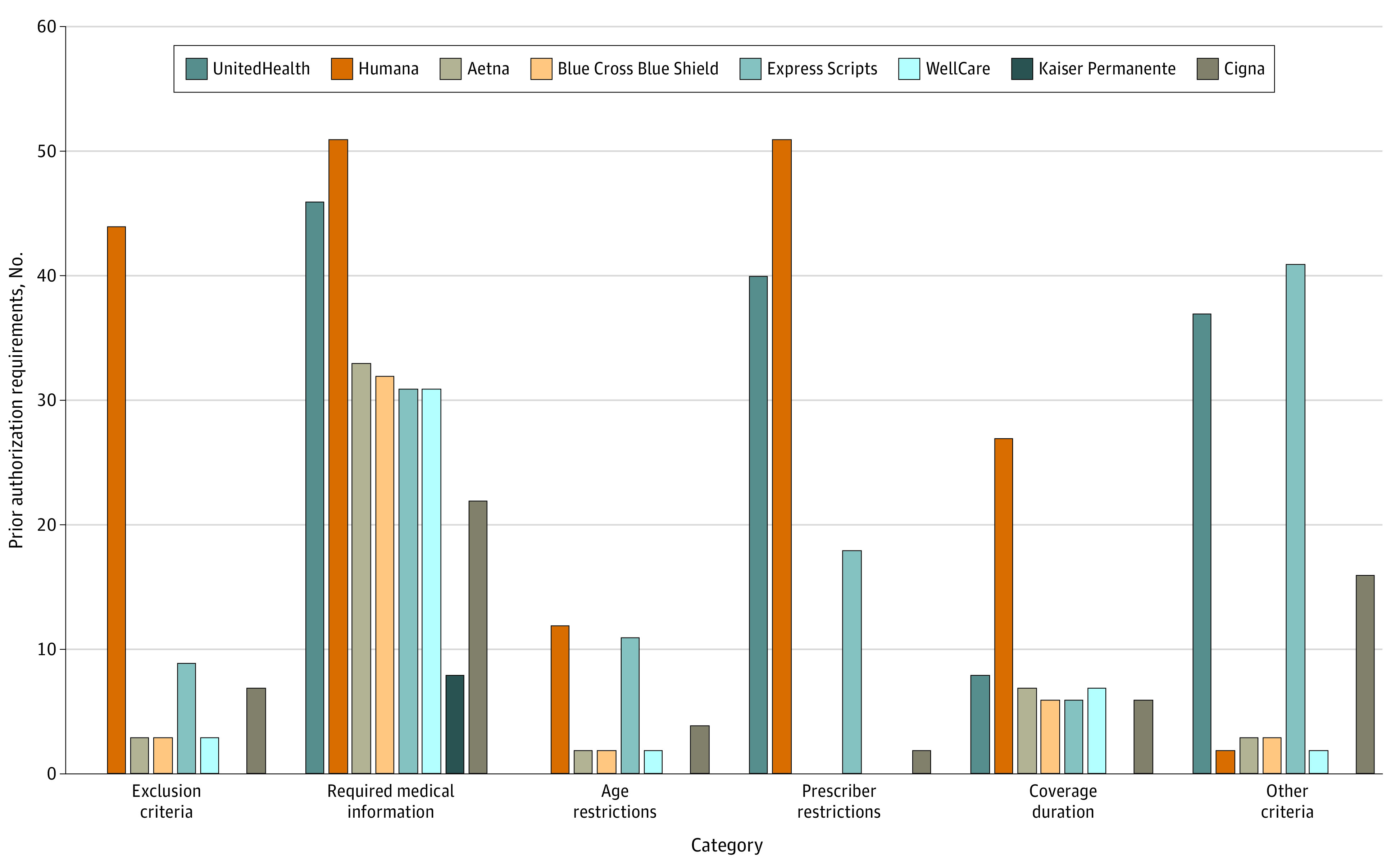
Frequency and Type of Prior Authorization Programs Covering New Drugs in Medicare Part D Part D plans offered by the 8 largest insurers in 2020 were identified in Cambridge, Massachusetts (zip code 02139) or Los Angeles, California (zip code 90029).

Forty-three (40%) drugs were covered by at least 1 of the largest plans with PA more restrictive than the approved labeling. Eighteen had shorter coverage duration than the full plan year. For 10 drugs, coverage by at least 1 of the largest plans was conditional on previous or coadministration of certain other therapies. The Table includes selected examples of PA policies that qualified as being more restrictive than the labeling.

**Table.** Selected Examples of Drugs Covered in at Least 1 Large Plan Formulary With Restrictive Prior Authorization (PA)

Drug name and FDA-approved indication	Restrictive PA	Reason
Dimethyl fumarate		
Indicated for the treatment of relapsing forms of multiple sclerosis, to include clinically isolated syndrome, relapsing-remitting disease, and active secondary progressive disease, in adults.	“Other criteria” for 1 insurer’s PA policy listed the following: “requests for clinically isolated syndrome will be approved for avonex (interferon β-1a) and betaseron (interferon β-1b) only.”	FDA-approved indication covers clinically isolated syndrome.
Conjugated estrogens/bazedoxifene		
Indicated for treatment of the following conditions in women with a uterus: treatment of moderate to severe vasomotor symptoms associated with menopause and prevention of postmenopausal osteoporosis.	“Required medical information” for 1 insurer’s PA policy listed: “treatment of moderate to severe vasomotor symptoms associated with menopause: diagnosis of moderate-to-severe vasomotor symptoms associated with menopause and the member must have had previous treatment, intolerance or contraindication to an selective serotonin reuptake inhibitor (eg, citalopram, fluoxetine, paroxetine hydrochloride) or venlafaxine. Prevention of osteoporosis: for the prevention of osteoporosis in a member who is postmenopausal, and the member must have had previous treatment, intolerance, or contraindication to either alendronate or raloxifene.”	Having had previous treatment, intolerance, or contraindication to selective serotonin reuptake inhibitors is not aligned with either approved labeling or routine practice.
Droxidopa		
Indicated for the treatment of orthostatic dizziness, light-headedness or the “feeling that you are about to black out” in adult patients with symptomatic neurogenic orthostatic hypotension caused by primary autonomic failure (Parkinson disease, multiple system atrophy, and pure autonomic failure), dopamine beta-hydroxylase deficiency, and nondiabetic autonomic neuropathy. Effectiveness beyond 2 weeks of treatment has not been established. The continued effectiveness of droxidopa should be assessed periodically.	“Other criteria” for 1 insurer’s PA policy listed: “trial and failure, contraindication, or intolerance to 1 of the following agents: fludrocortisone acetate, midodrine.”	Although fludrocortisone is generally first-line treatment in practice, droxidopa could be a reasonable alternative to midodrine. Trial and failure, contraindication, or intolerance to fludrocortisone not mentioned in approved labeling.
Ivabradine		
To reduce the risk of hospitalization for worsening heart failure in adult patients with stable, symptomatic chronic heart failure with reduced left ventricular ejection fraction. For the treatment of stable symptomatic heart failure due to dilated cardiomyopathy in pediatric patients aged 6 mo or older.	“Required medical information” for 1 insurer’s PA policy listed: “chronic heart failure (CHF) (initial): diagnosis of CHF. Patient has New York Heart Association (NYHA) class 2, 3, or 4 symptoms. Patient has a left ventricular ejection fraction less than or equal to 35%. Patient is in sinus rhythm. Patient has a resting heart rate of greater than or equal to 70 beats per minute. One of the following: patient is on a β-blocker at a maximally tolerated dose, or patient has a contraindication or intolerance to β-blocker therapy. Patient has been hospitalized for worsening heart failure in the previous 12 mos. Trial and failure, contraindication, or intolerance to maximally tolerated doses of an antiotensin converting enzyme (ACE) inhibitor or angiotensin receptor blockers. Dilated cardiomyopathy (DCM) (initial): Diagnosis of heart failure due to DCM. Patient has NYHA class 2, 3, or 4 symptoms. Patient is in sinus rhythm. Patient has an elevated heart rate. Trial and failure, contraindication or intolerance to 1 of the following: (1) β blocker (eg, bisoprolol, metoprolol succinate extended release), (2) ACE inhibitor (eg, captopril, enalapril), or (3) diuretic agent (eg, spironolactone, furosemide).”	Hospitalization in the previous 12 mo not specified in FDA-approved indication.
Brexpiprazole		
Use as an adjunctive therapy to antidepressants for the treatment of major depressive disorder. Treatment of schizophrenia.	“Required medical information” for 1 insurer’s PA policy listed: “major depressive disorder: the member must have clinically diagnosed major depressive disorder and the member must have documentation of prior therapy, intolerance, or contraindication to aripiprazole and at least 1 antidepressant therapy (ADT) and brexpiprazole must be used as adjunctive or add-on treatment to ADT and not as monotherapy. Schizophrenia: the member must have clinically diagnosed schizophrenia and the member must have documentation of prior therapy, intolerance, or contraindication to aripiprazole and 1 of the following: risperidone, olanzapine, quetiapine, or ziprasidone.”	Prior therapy, intolerance, or contraindication to aripiprazole not specified in approved labeling.
Abaloparatide		
Indicated for the treatment of postmenopausal women with osteoporosis at high risk for fracture.	“Other” criteria for 1 insurer’s PA policy listed: “treatment of postmenopausal osteoporosis, approve if the patient meets 1 of the following criteria: patient has tried 1 oral bisphosphonate or cannot take an oral bisphosphonate because the patient cannot swallow or has difficulty swallowing or patient cannot remain in an upright position after oral bisphosphonate administration or patient has a preexisting gastrointestinal medical condition (eg, patient with esophageal lesions, esophageal ulcers, or abnormalities of the esophagus that delay esophageal emptying [stricture, achalasia]), or patient has tried an intravenous bisphosphonate (ibandronate or zoledronic acid), or patient has severe kidney impairment or chronic kidney disease, or patient has had an osteoporotic fracture or fragility fracture.”	FDA-indication not restricted to patients with previous failed therapy. Abaloparatide can be first-line therapy for some patients in practice.
Venetoclax		
Indicated for the treatment of adult patients with chronic lymphocytic leukemia or small lymphocytic lymphoma. In combination with azacitidine or decitabine or low-dose cytarabine for the treatment of newly-diagnosed acute myeloid leukemia in adults who are aged 75 y or older, or who have comorbidities that preclude use of intensive induction chemotherapy. This indication is approved under accelerated approval based on response rates. Continued approval for this indication may be contingent on verification and description of clinical benefit in confirmatory trials.	“Required medication information” for 1 insurer’s PA policy listed: “chronic lymphocytic leukemia (CLL): the member has a diagnosis of CLL with or without deletion of 17p and 1 of the following applies: has received at least 1 prior therapy and the member is using venetoclax as monotherapy or in combination with rituximab or the request is for first-line therapy and the member is using venetoclax in combination with obinutuzumab. Acute myeloid leukemia (AML): the member has a diagnosis of newly-diagnosed AML and 1 of the following applies: member is aged 75 years or older or member has comorbidities that preclude the use of intensive induction chemotherapy (eg, baseline Eastern Cooperative Oncology Group performance status of 2-3, severe cardiac or pulmonary comorbidity, moderate hepatic impairment, or creatinine clearance less than 45 mL/min). The member will be using ventoclax in combination with azacitidine, or decitabine, or low-dose cytarabine.”	FDA-approved indication does not specify use of rituximab. Venetoclax can be used in combination with other drugs, such as obinutuzumab or ibrutinib after a patient has received at least 1 prior therapy.
Detetrabenazine		
Indicated for the treatment of chorea associated with Huntington disease. Tardive dyskinesia in adults.	“Other” criteria for 1 insurer’s PA policy listed: “chorea associated with Huntington disease: trial and failure, contraindication, or intolerance to tetrabenazine.”	Previous use of tetrabenazine not part of FDA labeling.

Abbreviation: FDA, US Food and Drug Administration.

## Discussion

In this cross-sectional study of drugs approved 2013 to 2017, 40% had PA criteria that placed conditions on formulary coverage beyond the FDA indication. Specific criteria varied considerably between insurers, which may increase the administrative burden on clinicians and beneficiaries seeking to consider PA policies when choosing among Part D plans or switching between insurers. More consistency in how plans implement PA in Part D could improve the experiences of patients and clinicians.^5^

Because we focused on new drugs approved between 2013 and 2017, our findings may not be generalizable to drugs approved in other periods. Another limitation is that our categorization of prior authorization criteria was conservative and may have underestimated the frequency of restrictive coverage requirements in Medicare Part D.

The consistency of PA requirements with the FDA labeling should be closely scrutinized. Because the FDA often extrapolates from clinical trials to approved indications,^6^ payers may be trying to ensure that patients receiving these drugs are more consistent with those in the pivotal trials.

## Notes

Supplement 1. eAppendix.

Supplement 2. Data Sharing Statement.
