# Supplementary material for: Characteristics of Prior Authorization Policies for New Drugs in Medicare Part D
Source: JAMA Health Forum. 2023 Feb 24;4(2):e225610. doi: 10.1001/jamahealthforum.2022.5610 (PMC9958521; doi:10.1001/jamahealthforum.2022.5610)
Supplement: Supplement 1. — eAppendix. [file jamahealthforum-e225610-s001.pdf]

Supplemental Online Content

Naci H, Forrest R, Zhai M, Stofesky AR, Kesselheim AS. Characteristics of prior authorization policies for new drugs in Medicare part D. *JAMA Health Forum*. 2023;4(2):e225610.
doi:10.1001/jamahealthforum.2022.5610

eAppendix.

This supplemental material has been provided by the authors to give readers additional information about their work.

© 2023 Naci H, et al. *JAMA Health Forum*.

eAppendix

We extracted information from PA documentation on the following categories: (1) exclusion criteria (factors that may disqualify patient eligibility for coverage such as use of concomitant treatments or stage of illness); (2) required medical information (criteria to ensure treatment is medically appropriate for a given patient, including the patient's clinical diagnosis and laboratory results); (3) age restrictions (whether certain age groups are not eligible for treatment); (4) prescriber restrictions (whether only certain medical specialists can prescribe the drug); (5) coverage duration limitations; and (6) other miscellaneous criteria.

To be conservative, we determined the consistency of PA characteristics with routine clinical practice, and consulted guidelines to identify the latest diagnostic criteria, treatment pathways, and contraindicated treatments that may not have been mentioned in the FDA labelling. For example, we considered insurer policies limiting the use of palbociclib alongside abemaciclib or ribociclib to be consistent with routine practice, as using these drugs in combination would be unlikely in clinical settings. Similarly, insurer policies that required very specific diagnostic criteria to determine patient eligibility for pirferidone were not categorized as restrictions, as such criteria were judged to be consistent with clinical practice guidelines.

We noted if coverage duration was for a shorter period than full plan year unless the drug was specifically indicated for short-term use or if the FDA labelling included a statement about lack of long-term data. For example, droxidopa's labelled indication stated that "effectiveness beyond 2 weeks has not been determined." Therefore, when Part D plans had limited coverage durations for this drug, we did not consider these conditions to be restrictive. We also determined if prior authorization policies were aligned with eligibility criteria of studies reported in Section 14 ('Clinical Studies') of the labelling.

© 2023 Naci H, et al. *JAMA Health Forum*.
