# Supplementary material for: Characteristics of Prior Authorization Policies for New Drugs in Medicare Part D
Source: JAMA Health Forum. 2023 Feb 24;4(2):e225610. doi: 10.1001/jamahealthforum.2022.5610 (PMC9958521; doi:10.1001/jamahealthforum.2022.5610)
Supplement: Supplement 2. — Data Sharing Statement. [file jamahealthforum-e225610-s002.pdf]

# Data Sharing Statement

Naci. Characteristics of Prior Authorization Policies for New Drugs in Medicare Part D. *JAMA Health Forum*. Published February 24, 2023. doi:10.1001/jamahealthforum.2022.5610

**Data**
**Data available:** No

**Additional Information**
**Explanation for why data not available:** No additional data are available.
